# Availability and accessibility of HIV self-tests and self-sample kits at community pharmacies in the Netherlands

**DOI:** 10.1186/s12981-023-00529-9

**Published:** 2023-06-22

**Authors:** Chaima Kandil, Jacqueline Hugtenburg, Titia Heijman, Hanna Bos, Martina Teichert, Renee Finkenflügel, Eline Op de Coul

**Affiliations:** 1 National Institute for Public Health and the Environment, Center for Infectious Disease Control, P.O. Box 1, Bilthoven, 3720 BA the Netherlands; 2 Amsterdam University Medical Center, Location VUMC, Amsterdam, the Netherlands; 3 Public Health Service of Amsterdam, Amsterdam, the Netherlands; 4 STI Aids Netherlands, Amsterdam, the Netherlands; 5 Royal Dutch Pharmacists Association (KNMP), the Hague, the Netherlands; 6 Public Health and Primary Care, Leiden University Medical Center, Leiden, the Netherlands; 7 Dutch Association of People with HIV (the HIV vereniging), Amsterdam, the Netherlands

**Keywords:** Pharmacies, HIV self-tests, HIV self-sampling, HIV testing, Home testing

## Abstract

**Background:**

In 2016 the WHO declared HIV self-testing and self-sampling an effective and safe test option that can reduce testing barriers. HIV self-tests and self-sampling kits (HIVST/HIVSS) are available for purchase at Dutch community pharmacies since 2019. We investigated the availability and accessibility of HIVST/HIVSS in community pharmacies, and factors associated with test availability.

**Methods:**

An online survey among all Dutch community pharmacies (n = 1,987) was conducted between April and June 2021. Availability of HIVST/HIVSS and experiences of pharmacists with the test offer were analyzed with descriptive statistics. The association of pharmacy and pharmacists’ characteristics with HIVST/HIVSS availability was explored by logistic regression analysis.

**Results:**

In total, 465 pharmacists completed the questionnaire. Of the responding pharmacists, 6.2% (n = 29) offered HIVST/HIVSS. The majority (82.8%) sold between 0 and 20 tests per year. In total, pharmacies sold an estimated 370 HIVST/HIVSS per year. Pharmacies having HIVST/HIVSS available were less often located in moderately-urbanized to rural neighborhoods (OR 0.35, 95%CI 0.16–0.77 versus highly-urbanized), and were less often located in moderate-to-low SES neighborhoods (OR 0.40, 95%CI 0.18–0.88 versus high-SES). Reasons for not offering HIVST/HIVSS by pharmacists were no or little demand (69.3%), and not being familiar with these tests (17.4%). 52% of the pharmacists provided information about testing to test buyers. Reported options to improve the test offer were giving advice about (performing) the test to test buyers (72.4%), placing tests visible on the counter (51.7%), and advertisement (37.9%).

**Conclusion:**

HIVST/HIVSS have a limited practical availability in Dutch community pharmacies since their introduction in 2019, especially in lower-urbanized and lower-SES areas. Further research is needed to explore how to expand access to HIVST/HIVSS through community pharmacies in the Netherlands, and how to tailor it to the needs of pharmacy clients.

## Introduction

Increasing access to HIV testing is crucial to achieve the Joint United Nations HIV/AIDS Programme 95-95-95 aims for 2025 for HIV testing, treatment and viral suppression, and the World Health Organization’s (WHO) 2030 elimination goals for HIV [1–3]. Especially in the post COVID-19 era, when healthcare systems continue to be impacted and budgets for sexual healthcare are tight, alternative test approaches become even more important [4–6].

In 2016, the WHO declared HIV self-testing and self-sampling a safe and effective test option that offers convenience for priority populations that may not test otherwise [7]. In 2018, a strategic framework to support countries to implement and scale up HIV self-testing was published by the WHO [8]. HIV self-testing or -sampling allows an individual to collect their own oral fluid or blood specimen to conduct the test in a private setting, and to independently interpret the result themselves (self-testing) or to send their sample to a laboratory that returns the test result to the individual (self-sampling) [3, 8, 9]. Both HIV self-testing and self-sampling are highly acceptable, easy to use, and successful in reaching populations at higher risk for HIV, and first-time testers [10]. Testing at home could also overcome testing barriers, such as confidentiality and privacy concerns, fear for stigma and discrimination, and accessibility issues at healthcare providers such as waiting lists at sexual health centers (SHCs), waiting times for test results, and high costs (deductible) for HIV testing at general practices (GP) [10–13].

Normalizing and promoting HIV testing, whether in a healthcare setting, at home, or through peers, are part of the Dutch national action plan for STI, HIV and sexual health for 2023–2027. HIV self-tests and self-sampling kits (HIVST/HIVSS) are available for purchase at Dutch community pharmacies since 2019 and through online (commercial) providers since 2017, at a price range of €15–35. HIV self-tests offered in the Netherlands include finger prick blood tests and oral fluid tests. Most HIV self-sampling kits use finger prick blood.

Community pharmacies are community-centered and easily accessible; it is in this context that community pharmacists and their staff could play a role in advocating and implementing prevention programs, such as the provision of HIVST/HIVSS. The more so since the pharmacist is generally regarded as a reliable and knowledgeable healthcare provider (HCP), who is experienced in conveying information and advice to pharmacy clients. Currently, it is unknown how many pharmacies in the Netherlands have HIVST/HIVSS available, how pharmacists experience the test offer, including their vision on its accessibility for clients. This information is important to inform the Dutch HIV prevention and -testing policy, and to develop pharmacy services regarding HIVST/HIVSS. International research on HIVST/HIVSS provided by pharmacies is also rather limited [14–19] or studies focus on rapid point-of-care HIV testing conducted by pharmacists [20–29].

The aim of our study was to explore the availability and accessibility of over-the-counter (OTC) HIVST/HIVSS in Dutch community pharmacies, factors associated with HIVST/HIVSS availability, and experiences of pharmacy staff with the test provision.

## Methods

### Study design and setting

For this cross-sectional study, an email invitation that included research goals, an informed consent, and a link to an online survey, was send to all community pharmacies (n = 1,987) in the Netherlands. Pharmacy members of the Royal Dutch Pharmacists Association (‘Koninklijke Nederlandse Maatschappij ter bevordering der Pharmacie’, KNMP) received this study invitation through a KNMP mailing list that included general email addresses of all pharmacies. A first reminder email was send 21 days after the initial invitation. To enhance the response rate, a second reminder, using a mailing list with personal email addresses of pharmacists, was send 21 days after the first reminder. Data were collected between April 24th and June 14th 2021.

### Data collection and analyses

The questionnaire was developed and pre-tested by a team of researchers from the National Institute for Public Health and the Environment (RIVM), the VU university (‘Vrije Universiteit’) in Amsterdam, the Public Health Service of Amsterdam (GGD Amsterdam), and the KNMP, including two epidemiologists, an anthropologist, two pharmacists, a KNMP data analyst, and a medical officer working for the Dutch association of people with HIV.

The questionnaire included 43 questions (pre-coded or open) comprising the topics of (1) pharmacy and pharmacist characteristics, (2) availability and accessibility of HIVST/HIVSS in pharmacies, and (3) pharmacists’ experiences with providing HIVST/HIVSS. Data collected from pharmacists included age, gender, highest education level, profession (pharmacist/pharmacist in training/other), employment status (full-time/part-time), years of work experience, postal code area of the pharmacy, staffing to support the service (number of pharmacists and number of employees working at the same time on a daily basis), type of HIVST/HIVSS (oral fluid self-test/finger prick self-test/finger prick self-sample kit), brand of the test, experiences with providing HIVST/HIVSS to clients, information provided to clients who buy a test, privacy of clients who have questions about HIV testing, and impressions of pharmacists on the characteristics of test buyers (gender, Western/non-Western migration background, first-time buyers/repeat buyers).

Questionnaire data (anonymous) were downloaded from Questback and entered into an SPSS database (IBM SPSS Statistics for Windows, version 28, IBM Corp, Armonk, New York) at the RIVM. Data on socioeconomic status (SES) and level of urbanisation (OAD: ‘Omgevingsadressendichtheid’) were merged with the SPSS database, based on postal code area (four digits) of the pharmacy. SES scores were obtained from the Netherlands Institute for Social Research (www.scp.nl). SES scores included classes SES 1 (strongly prosperous) to SES 5 (strongly deprived). The score takes into account the average income per household in the postal code area, as well as the percentage of households with low income, without paid work, and with low education level. The level of urbanisation by postal code area was obtained from Statistics Netherlands (www.cbs.nl). Urbanisation contained the classes OAD 1 (very highly urban) to OAD 5 (rural).

Descriptive statistics were used for explorative analyses. Univariable and multivariable logistic regression analyses were conducted with the variables of gender, age, work experience, number of employees, SES, level of urbanisation, and province for associations with the outcome variable of pharmacies offering HIVST/HIVSS (yes/no). In case of small numbers, categories were merged. Variables with p-values of p < 0.2 were included in the multivariable model. Significantly correlated variables (Spearman correlation coefficients) were not included in the model together. Odds ratios (OR) and 95%CI were computed. Variables were considered statistically significant when p < 0.05 for the likelihood ratio test (backward selection).

## Results

### Response rate and characteristics of pharmacists

In total, 1,987 community pharmacies included in the KNMP database received the first email invitation and questionnaire. From these, 118 (5.9%) pharmacists completed the questionnaire. After the reminder, a total of 324 questionnaires were received (response rate: 16.3%). To further enhance the response rate, a second reminder was send to a broader mailing list of the KNMP (n = 3,107) including personal email addresses of pharmacists from these 1,987 community pharmacies. An extra 141 pharmacists participated, resulting in a total of 465 completed questionnaires. This corresponded to an estimated 20.0-23.4% response rate for community pharmacies, by using the number of 4-digit postal codes (n = 397, 20.0%) as an indication for the minimum number of unique participating pharmacies. As the 4-digit postal code areas may include more than one pharmacy in population dense areas, the estimated maximum number of unique participating pharmacies was 465 (23.4%).

Baseline characteristics of the survey participants are shown in Table 1. The median age of the participants was 43 years (interquartile range, IQR, 34–55 years), 57.2% were female, and 97.8% were pharmacist or pharmacist in training. Ten participants (2.2%) were pharmaceutical consultant or pharmacy assistant. Of the participants, 95.7% were working at community pharmacies, 4.1% at outpatient pharmacies, and 0.2% at an online pharmacy. The median number of years of working experience was 18 (IQR 8–26 years). The median number of pharmacists per pharmacy was two.

**Table 1:** Baseline characteristics of pharmacists and pharmacies

HIV self-tests/-sample kits offered at pharmacies (N,%)	Yes 29 (6.2)	No 436 (93.8)	Total 465 (100)
Gender (N,%)	29	431	460
Male	16 (57.1)	181 (41.9)	197 (42.8)
Female	12 (42.9)	251 (58.1)	263 (57.2)
Age (years, median, IQR)	40 (34-48.5)	44 (34–55)	43 (34–55)
Profession	29	433	462
Pharmacist	27 (93.1)	408 (94.2)	435 (94.2)
Pharmacist in training	2 (6.9)	15 (3.5)	17 (3.7)
Pharmaceutical Consultant/Pharmacy Assistant	0 (0)	10 (2.3)	10 (2.2)
Highest education	28	433	461
University or higher vocational education	27 (96.4)	414 (95.6)	441 (95.7)
Other education	1 (3.6)	19 (4.4)	20 (4.3)
Employment status	29	428	457
Full-time	19 (65.5)	302 (70.6)	321 (70.2)
Part-time	10 (34.5)	126 (29.4)	136 (29.8)
Work experience (years, median, IQR)	15 (7-23.5)	18 (8–26)	18 (8–26)
Pharmacy type	29	436	465
Community pharmacy	27 (93.1)	418 (95.9)	445 (95.7)
Outpatient pharmacy	1 (3.4)	18 (4.1)	19 (4.1)
Online pharmacy	1 (3.4)	0 (0)	1 (0.2)
Nr of pharmacists per pharmacy (median, IQR)	2 (1–2)	2 (1–2)	2 (1–2)
Nr of employees working at 1 day	28	435	463
0–4	11 (39.3)	97 (22.3)	108 (23.3)
5 or more	17 (60.7)	338 (77.7)	355 (76.7)
Region (Province) of pharmacy (N,%)	29	436	465
West (N-Holland, Z-Holland, Utrecht, Zeeland)	18 (62.1)	222 (50.9)	240 (51.6)
South (Noord-Brabant, Limburg)	7 (24.1)	86 (19.7)	93 (20.0)
East (Flevoland, Overijssel, Gelderland)	4 (13.8)	92 (21.1)	96 (20.6)
North (Groningen, Friesland, Drenthe)	0 (0.0)	36 (8.3)	36 (7.7)
Pharmacy in city or village (N,%)	29	436	465
City	26 (89.7)	298 (68.3)	324 (69.7)
Village	3 (10.3)	138 (31.7)	141 (30.3)
Level of urbanisation (N,%)	29	436	465
Highly urbanized (OAD 1)	14 (48.3)	112 (25.7)	126 (27.1)
Moderately urbanized (OAD 2–4) to rural (OAD 5)	15 (51.7)	324 (74.3)	339 (72.9)
SES (N,%)	29	436	465
High (SES 1–2)	16 (55.2)	153 (35.1)	169 (36.3)
Moderate (SES 3–4) to low (SES 5)	13 (44.8)	283 (64.9)	296 (63.7)

IQR: Interquartile range, N-Holland: Noord-Holland, Z-Holland: Zuid-Holland, SES: socioeconomic status, OAD: ‘Omgevings Adressen Dichtheid’ (Level of urbanisation)

Twenty-nine pharmacists (6.2%) at 29 unique pharmacies reported to sell HIVST/HIVSS. Pharmacies selling tests were mostly (71.6%) located in the West region of the Netherlands where the major cities Amsterdam, Rotterdam, the Hague, and Utrecht are located. Also pharmacies in the South region (provinces Noord-Brabant and Limburg) are selling HIVSS/HIVST, but no pharmacies in the North region (Groningen, Friesland, Drenthe).

### Factors associated with availability of HIV self-tests and -sampling kits at pharmacies

Univariable regression analyses showed that the variables: number of employees working at the same time on a daily basis in the pharmacy, pharmacy location (city/village), urbanisation of pharmacy area, and SES of pharmacy area, were significantly associated with the availability of HIVST/HIVSS (Table 2). After adjustment for these covariates, the variables level of urbanisation and SES of pharmacy area remained in the model. Community pharmacies having HIVST/HIVSS available were less often located in moderately urbanized to rural areas (0.35; 95%CI 0.16–0.77 versus highly urbanized), and in moderate to low SES neighborhoods (OR 0.40; 0.18–0.88 versus high SES) compared to pharmacies not having HIVST/HIVSS available.

**Table 2:** Factors associated with pharmacies having HIV self-tests and -self-sampling kits available

	Crude OR (95%Cl)	p-value	Adjusted OR^1^ (95%CI)	p-value
Gender of pharmacist				
Male	1			
Female	0.54 (0.25–1.17)	0.12	ns	
Age of pharmacist				
24–44 years	1			
45–69	0.75 (0.35–1.61)	0.46	na	
Work experience (years)				
0–10	1			
11 or more	0.64 (0.30–1.37)	0.25	na	
Nr of employees (1 day)				
0–4	1			
5 or more	**0.44 (0.20–0.98)**	**0.04**	ns	
Region of pharmacy				
West	1			
South	1.00 (0.41–2.49)	0.99	na	
East	0.54 (0.18–1.63)	0.27		
North	na			
City or village				
City	1			
Village	**0.25 (0.07–0.84)**	**0.03**	na	
Urbanisation of pharmacy neighborhood				
Highly urban	1		1	
Moderately urban to rural	**0.37 (0.17–0.79)**	**0.01**	**0.35 (0.16–0.77)**	**0.01**
SES of pharmacy neighborhood				
High	1		1	
Moderate to low	**0.44 (0.21–0.94)**	**0.03**	**0.40 (0.18–0.88)**	**0.02**

1. Multivariable model adjusted for age, gender, and number of employees working in the pharmacy at the same time at one day. Number of years of work experience is not included in the multivariable model due to correlation with age (Correlation Coefficient: 0.66, p < 0.001). City/village is not included in the multivariable model due to correlation with level of urbanisation (Correlation Coefficient: 0.34, p < 0.001). Adjusted by backward stepwise method. Significant associations are shown in bold. OR: odds ratios; CI: confidence interval; ns: not significant; na: not applicable

### Availability and accessibility to HIV self-tests and -sampling kits

Of the 29 community pharmacies offering HIVST/HIVSS, 24 (82.8%) sold between 0 and 20 tests per year, four pharmacies (13.8%) between 21 and 40 tests, and one (3.4%) more than 41 tests per year (Table 3). In total, these pharmacies sold an estimated 370 HIVST/HIVSS per year.

**Table 3:** Responses of pharmacists offering HIV self-tests and self-sampling kits

Type of test*	N = 29 (%)
Oral fluid HIV self-test	4 (13.8)
Fingerstick HIV self-test	15 (51.7)
Fingerstick HIV self-sample kit	15 (51.7)
Other	3 (10.3)
Brand of test*	N = 29 (%)
INSTI®	10 (34.5)
ByMe®	6 (20.7)
SPO® (SOA poli online)	15 (51.7)
Autotest HIV®	1 (3.4)
On order	2 (6.8)
**Demand for HIV tests by clients**	N = 29 (%)
No, never	8 (27.6)
Yes, sometimes	17 (58.6)
How can pharmacists contribute to promotion of HIV tests*	N = 29 (%)
Not	4 (13.8)
Visible to the consumer	15 (51.7)
Pointing out the test to clients who come for different purpose	6 (20.7)
Advertisement	11 (37.9)
Advice to clients who come for the test	21 (72.4)
**How many tests sold per year?**	N = 29 (%)
0–20	24 (82.8%)
21–40	4 (13.8%)
41 or more	1 (3.4)
**Main customers**	N = 29 (%)
Mostly men	17 (58.6)
Mostly women	0 (0)
Both about the same	2 (6.9)
No opinion	10 (34.5)
	N = 29 (%)
Mostly people with a Western or no migration background	14 (48.3)
Mostly people with a non-Western migration background	0 (0)
Both about the same	3 (10.3)
No opinion	12 (41.3)
	N = 29 (%)
Mostly first-time testers	3 (10.3)
Mostly frequent testers	4 (13.8)
Both about the same	8 (27.6)
No opinion	14 (48.3)
**HIV test preference of pharmacist**	N = 29 (%)
HIVSS	12 (41.4)
HIVST (oral)	2 (6.9)
HIVST (finger prick)	5 (17.2)
No preference	9 (31.0)
Other	1 (3.4)

*Percentages do not add up to 100, due to multiple possible answers

Most pharmacies (65.5%, n = 19) had one type of HIVST/HIVSS in stock, 27.6% (n = 8) two or three different tests, and 6.9% (n = 2) had no tests in stock, but tests were ordered on demand. The fingerstick INSTI® (34.5%) and ByME® (20.7%) HIVST, and the Soapoli-online.nl (SPO®) (51.7%) HIVSS were the most offered HIV tests at pharmacies. Four pharmacies (13.8%) were (also) offering oral fluid HIVST.

Most pharmacies (89.7%) kept the HIVST/HIVSS behind the counter. Only 10.3% kept the tests in shelves, visible for the customer. These pharmacies were all, except one, located in the West and South of the Netherlands. Also, pharmacies in these areas sold larger numbers of tests compared to pharmacies located in other regions, and had a larger assortment of HIVST/HIVSS in stock.

Of pharmacists offering HIVST/HIVSS, 79.3% never recommended tests to clients who visit the pharmacy, and 58.6% indicated an occasional demand for HIVST/HIVSS. Pharmacists saw options to improve the test offer: 51.7% indicated that they could contribute to the promotion of HIVST/HIVSS by giving advice about the test to test buyers (72.4%), by placing the tests visible to pharmacy clients, and by advertisement (37.9%).

In 48.3% of the pharmacies, the price of HIVST/HIVSS was between 25 and 35 euro, in 31.1% between 35 and 45 euro, and in 6.9% above 45 euro. 13.7% of the pharmacists didn’t know the price. The profit margin on one sold test was between 5 and 10 euros in 44.8% of the pharmacies, between 10 and 15 euro in 10.3% of the pharmacies, and 44.8% of the pharmacists didn’t know the profit margin. In total, 20.7% of the pharmacists were willing to dispense HIVST/HIVSS to clients at no profit, although 52% didn’t believe that a price reduction will lead to an increase in sales.

Pharmacists not offering HIVST/HIVSS were asked for their main reasons; the majority (69.3%) indicated that there is no or little demand for tests, 17.4% were not familiar with HIVST/HIVSS, 3.2% did not know, and 10.1% reported other reasons through an open question, such as: ‘’*due to the correct interpretation of test results, we think it would be better to have this type of testing carried out by healthcare professionals’’.* Or: *“I think this is primarily a task for the GP”.* Another pharmacist described: *‘’given the seriousness of the condition [HIV], we deliberately do not offer this; a false positive or false negative result affects the patient’’.* Other reasons of pharmacists were that there is no clarity about the reliability of the tests, that the tests were not in the product range of their pharmacy chain, or *‘’there is no KNMP policy’’.*

### Pharmacists’ experiences with HIV self-tests and -sampling kits

According to 58.6% (n = 17) of the 29 pharmacists that have HIVST/HIVSS available, test buyers are mostly men, 6.9% thought the gender distribution was similar, but 34.5% had no opinion about gender distribution (Table 3). In addition, 48.3% indicated that the main customers are people with a Western or no migration background, 10.3% indicated a similar distribution of clients with a Western vs. non-Western background, and 41.4% did not know. Lastly, frequent testers for HIV were the main customers according to 13.8% of the pharmacists, 10.3% reported first-time testers as main customers, 27.6% indicated this was similar, and 48.3% had no opinion.

Of the 29 pharmacists offering HIVST/HIVSS, 28 answered questions about received training on HIV tests. Half (n = 14) received some training; mostly about how to conduct the HIV test (n = 14), what to do in case the test is positive (n = 10), the window phase (n = 7), wrong test results (n = 5), HIV risks (n = 5), and HIV treatment (n = 4). Although only half of the pharmacists received some training on HIVST/HIVSS, 96% (n = 27) felt adequately or well prepared to provide these tests. Fifteen pharmacists (51.7%) informed test buyers about HIV follow-up and -prevention, four (13.8%) about HIV treatment, twelve (41.4%) about HIV/STI test locations, and nine (31.0%) about Pre-Exposure Prophylaxis (PrEP). At 5 of the 29 pharmacies (17.2%), test results were occasionally reported back by the client.

Most pharmacists (41.4%, n = 12) offering HIVST/HIVSS preferred HIVSS over HIVST (Table 3), 9 (31.0%) had no preference for type of test, 7 (24.1%) preferred HIVST (5 finger prick, 2 oral). Pharmacists selling HIVSS provided more information to customers compared to pharmacists selling HIVST. One pharmacist reported to prefer HIVSS over HIVST as clients receive follow-up care and referral to an appropriate HCP.

## Discussion

The percentage of community pharmacies having HIV self-tests and -self-sampling kits (HIVST/HIVSS) available was low (6.2%) in the Netherlands, and most pharmacies only sold small numbers (0–20) per year. Pharmacies offering HIVST/HIVSS were more often located in urban areas, and in neighborhoods of medium to high SES. Reasons for not selling HIV tests were no or little demand, not being familiar with the tests, concerns about test reliability, or opinions about clients being better off by testing for HIV at the GP or other healthcare provider.

This is the first nationwide study on availability and accessibility of HIVST/HIVSS at community pharmacies in the Netherlands. The response rate among pharmacies was 20–23%, which is higher than previous surveys conducted through the KNMP with response rates between 15 and 20% [30]. Our study also has some limitations. First, it is unknown whether the ratio of participating pharmacies selling or not selling HIVST/HIVSS is generalizable to all community pharmacies in the Netherlands. Therefore, for data validation, we also obtained pharmacy dispensing data on HIVST/HIVSS from the Dutch Foundation for Pharmaceutical Statistics (SFK, ‘Stichting Farmaceutische Kengetallen’, www.sfk.nl). The SFK database contains data from > 95% of the community pharmacies in the Netherlands, and covers around 15.8 million people (on a population of 17.9 million, dec 2021, www.cbs.nl). We compared the estimated number of distributed HIVST/HIVSS per year from our survey (n = 370) with the yearly number of HIVST/HIVSS dispensations in the SFK registry, which showed similar results: 300–400 per year in 2019–2021, suggesting that most pharmacies offering HIVST/HIVSS participated in our survey, and may have been overrepresented in our survey compared to pharmacies not selling HIVST/HIVSS. Furthermore, the number of registered HIVST/HIVSS dispensations in this registry is likely an underreport of all HIVST/HIVSS dispensations of community pharmacies in the Netherlands, as the SFK does not receive data from all pharmacies, but for about 85%, for over-the-counter (OTC) products, and continuity of OTC data reporting over time is unknown [personal communication, Jeroen Lukaart, SFK]. Although the level of underreporting of HIVST/HIVSS dispensations in the SFK database is uncertain, this data confirms our results that, nationwide, only small numbers of HIV tests are sold at Dutch pharmacies.

Another limitation of our study is that experiences of pharmacy assistants were not asked about, and opinions may differ between professions. However, we noticed that some pharmacy assistants filled in our questionnaire. The first email invitation was send to the general pharmacy email addresses, and pharmacy assistants may also had access to the survey. Finally, we did not collect information from test buyers, which appeared not practically feasible in the planned study period, also due to the low number of pharmacies selling HIVST/HIVSS, and the low numbers of tests distributed per year.

There are a few international studies published on HIVST/HIVSS provided by pharmacies [14–19]. A French study among pharmacies in Caen showed that 44% had HIV self-tests available for sale [15]. Another French study on opinions of pharmacists and (potential) users of HIV self-tests, including men who have sex with men (MSM) and migrants from sub-Saharan Africa, showed that all participants perceived the HIV self-test offer by pharmacists as a significant step forward to improve access to HIV testing [14]. However, sales prices, concerns about how to use the test, interpret results correctly, and anonymity were seen as obstacles to HIV self-testing through pharmacies in France [14]. A survey among 361 New York pharmacies [16] revealed that HIV self-test kits were available in 27% and accessible in 10% of the pharmacies, and there was no difference between pharmacies in neighborhoods with high or low HIV diagnoses rates. The reason that Dutch pharmacies selling HIVST/HIVSS are more often located in urban areas could be explained by the fact that populations at risk of HIV are more concentrated in those areas [31]. In the Netherlands, the main HCP for HIV testing are GPs and SHCs, and rates of HIV self-testing or - home-sampling are still low. In a 2018 survey conducted among MSM in the Netherlands, 0.5% used an HIVSS and 0.5% an HIVST the last time they tested (personal communication, C. Den Daas, university of Aberdeen). However, 6.7% would prefer to use an HIVST and 3.1% an HIVSS. In the MSM survey, it was not asked where the HIVST/HIVSS was purchased from, but community pharmacies likely played a minor role [32]. Probably also because STI and HIV testing at SHCs in the Netherlands is free of charge for populations at risk including MSM. A study conducted among MSM in five European countries, which assessed HIV self-testing in the context of the main HIV test providers, showed that self-testing was the preferred testing option for 34% of the participants [33]. A Spanish study among MSM who never tested for HIV, concluded that HIV self-testing has the potential of becoming a highly-used testing methodology. Of the 2,589 MSM included in this study, 83% would have used self-testing if it was available [34]. Although HIVST/HIVSS are available in the Netherlands, results from the Dutch MSM survey may indicate a lack of familiarity with HIVST/HIVSS. HIV self-testing or -sampling may empower individuals by strengthening self-care and self-reliance, and is a good alternative for those who prefer autonomous and anonymous testing [7]. It may also reduce practical testing barriers, especially in rural areas, where the distance to a SHC or other HIV test location is longer compared to urban areas. Travel distance is known to be inversely associated with SHC utilization [35]. In rural areas, a community pharmacy offering HIVST/HIVSS could therefore improve the test offer in the Netherlands. Although there is no KNMP policy on HIVST/HIVSS, this organization is the best gateway to promote the distribution of HIVST/HIVSS among pharmacies in those areas.

Some pharmacists indicated that HIV tests should preferably be administered by a HCP. However, several studies showed that the majority of users of HIV self-tests are quite capable to correctly interpret HIV test results [36], but follow-up care needs to be well-organized. HIV-self-testing or- sampling is meant as an extra test option for populations at high or low risk for HIV. People at low risk are not eligible for testing at the SHC, and due to capacity constraints people at high risk might not always have access to a SHC. Therefore, pharmacies can fill a gap for whom testing at the GP is a barrier. However, people may not know about the HIV test availability at pharmacies, and tests are often placed behind the counter. By placing the tests visible to the consumer, by advertisement, or by giving advice to clients who come for the test, awareness about HIV testing among pharmacy clients can be improved. In our study, pharmacists selling HIVSS provided more guidance compared to those selling HIVST, while the opposite was expected, as laboratories provide test result for HIVSS that increases the opportunity to guide people into care. People using HIVST have to seek follow-up care by themselves in case of a reactive result. When disseminating (and providing information about) HIVST/HIVSS, pharmacists should therefore provide information about the test and follow-up steps, including information about if the test result is reactive, a doctor should be consulted for confirmation testing and follow-up care. We also recommend training for pharmacists about HIVST/HIVSS, so that pharmacists subsequently provide correct information to customers who purchase an HIV test.

## Conclusion

Despite its availability since 2019, the HIVST/HIVSS has limited practical availability in Dutch community pharmacies in the Netherlands. However, pharmacists see opportunities to improve the HIV test offer. Additional research is needed to identify strategies to promote the uptake of and the availability of HIV tests in community pharmacies, for instance by conducting in-depth interviews among pharmacists, pharmacy technicians and clients. Also, if pharmacies are going to be leveraged as channel for providing HIVST/HIVSS, this should preferably involve pharmacy chains and the KNMP. Key populations at risk for HIV could be more informed about the availability and potential benefits (or drawbacks) of the various HIV tests at community pharmacies.

## Data Availability

The datasets generated are available through the corresponding author on reasonable request.
